# Mutation-Dependent Pathomechanisms Determine the Phenotype in the Bestrophinopathies

**DOI:** 10.3390/ijms21051597

**Published:** 2020-02-26

**Authors:** Anna-Lena Nachtigal, Andrea Milenkovic, Caroline Brandl, Heidi L. Schulz, Lisa M. J. Duerr, Gabriele E. Lang, Charlotte Reiff, Philipp Herrmann, Ulrich Kellner, Bernhard H.F. Weber

**Affiliations:** 1 Institute of Human Genetics, University of Regensburg, Franz-Josef-Strauß-Allee 11, 93053 Regensburg, Germany; anna-lena.nachtigal@klinik.uni-regensburg.de (A.-L.N.); andrea.milenkovic@klinik.uni-regensburg.de (A.M.); caroline.brandl@klinik.uni-regensburg.de (C.B.); lhmaier@gmail.com (H.L.S.); lisa-marie.duerr@klinik.uni-regensburg.de (L.M.J.D.); 2 Klinik und Poliklinik für Augenheilkunde, Universitätsklinikum Regensburg, Franz-Josef-Strauß-Allee 11, 93042 Regensburg, Germany; 3 Lehrstuhl für Genetische Epidemiologie, Universität Regensburg, Franz-Josef-Strauß-Allee 11, 93053 Regensburg, Germany; 4 Universitätsklinikum Ulm, Augenklinik, Prittwitzstraße 43, 89075 Ulm, Germany; gabriele.lang@uniklinik-ulm.de; 5 Medical Practice Stadttheater, Bertoldstr. 45, 79098 Freiburg im Breisgau, Germany; cpolo@gmx.de; 6 Universitäts-Augenklinik Bonn, Ernst-Abbe-Str. 2, 53127 Bonn, Germany; philipp.herrmann@ukbonn.de; 7 Zentrum für seltene Netzhauterkrankungen, AugenZentrum Siegburg, MVZ Augenärztliches Diagnostik- und Therapiecentrum Siegburg GmbH, Europaplatz 3, 53721 Siegburg, Germany; kellneru@mac.com; 8 RetinaScience, 53113 Bonn, Germany

**Keywords:** bestrophin-1, BEST1, induced pluripotent stem cell, retinal pigment epithelium, ER-associated degradation, endo-lysosomal degradation pathway, pathomechanism, Best vitelliform macular dystrophy, Best disease, autosomal recessive bestrophinopathy, autosomal dominant vitreoretinochoroidopathy

## Abstract

Best vitelliform macular dystrophy (BD), autosomal dominant vitreoretinochoroidopathy (ADVIRC), and the autosomal recessive bestrophinopathy (ARB), together known as the bestrophinopathies, are caused by mutations in the bestrophin-1 (*BEST1*) gene affecting anion transport through the plasma membrane of the retinal pigment epithelium (RPE). To date, while no treatment exists a better understanding of BEST1-related pathogenesis may help to define therapeutic targets. Here, we systematically characterize functional consequences of mutant BEST1 in thirteen RPE patient cell lines differentiated from human induced pluripotent stem cells (hiPSCs). Both BD and ARB hiPSC-RPEs display a strong reduction of BEST1-mediated anion transport function compared to control, while ADVIRC mutations trigger an increased anion permeability suggesting a stabilized open state condition of channel gating. Furthermore, BD and ARB hiPSC-RPEs differ by the degree of mutant protein turnover and by the site of subcellular protein quality control with adverse effects on lysosomal pH only in the BD-related cell lines. The latter finding is consistent with an altered processing of catalytic enzymes in the lysosomes. The present study provides a deeper insight into distinct molecular mechanisms of the three bestrophinopathies facilitating functional categorization of the more than 300 known *BEST1* mutations that result into the distinct retinal phenotypes.

## 1. Introduction

Bestrophin-1 (BEST1) belongs to the bestrophin family of four evolutionarily related genes (*BEST1–4*) that encode distinct integral membrane proteins [1]. In humans, they function as calcium-activated anion channels differing in expression regulation and tissue distribution [2,3]. The best characterized family member to date is BEST1 and localizes to the basolateral aspect of the retinal pigment epithelium (RPE) [4,5]. Mutations in the protein encoding gene are linked to at least three distinct retinopathies that can be distinguished by phenotype and mode of inheritance: The autosomal dominant Best vitelliform macular dystrophy (BD; MIM 153700) [6,7], the autosomal dominant vitreoretinochoroidopathy (ADVIRC; MIM 193220) [8], as well as the autosomal recessive bestrophinopathy (ARB; MIM 611809) [9]. While a typical phenotype presentation can be assigned to each of the distinct entities, a clear genotype-phenotype correlation is limited as individuals with BEST1-associated retinal defects present with a remarkable inter- and intra-familial phenotypic variability. Accordingly, genetically affected family members of patients with a severe form of the bestrophinopathies may even remain asymptomatic throughout their entire life [2,10]. An abnormal Arden ratio (light peak/dark trough ratio) in the electro-oculogram (EOG) response is regarded pathognomonic for the BEST1-associated clinical manifestations [11].

BD is the most frequent pathology of the bestrophinopathies, which classically begins in childhood and has an estimated prevalence between 1:5,000 [12] and 1:67,000 [13]. The disease predominantly affects the macula in the center of the retina and is characterized by an accumulation of fluid and yellow yolk-like lesions (vitelliform) beneath the neurosensory retina. The lesions evolve progressively over many years leading to a variable degree of vision impairment [14]. In contrast, ADVIRC is a very rare chorioretinal pigmentary condition affecting the peripheral retina with an estimated prevalence of 1:1,000,000 (https://www.orpha.net, accessed in November 2019). Up to now, only five *BEST1* mutations have been identified in ADVIRC (Human Gene Mutation Database (HGMD), http://www.hgmd.cf.ac.uk/ac/ and Leiden Open Variation Database v.3.0 (LOVD), https://databases.lovd.nl/shared/genes/BEST1; both accessed in November 2019). The disease is classically characterized by a peripheral retinal circumferential hyperpigmented band with a well-defined posterior demarcation and can be associated with developmental ocular anomalies such as microcornea, microphthalmos, angle closure glaucoma, and cataract [15]. In 2008, Burgess et al. first reported ARB as a distinct retinal entity associated with homozygous or compound heterozygous *BEST1* gene mutations [9,16,17]. The clinical presentation of ARB is unique and different from the classical BD phenotype as multifocal subretinal deposits are predominantly outside the macula area. Among the roughly 300 *BEST1* mutations known so far, only about 15% are associated with the ARB phenotype. Heterozygous parents generally show no retinal symptoms [16,17].

The discovery of the crystal structure of BEST1 orthologues [18,19], and the availability of electron microscopy and electrophysiological recordings revealed that the introduction of various mutations into the chicken BEST1 produces channels with dramatically altered gating properties and diminishes channel inactivation [20]. In cell culture model systems disease-causing *BEST1* mutations have been reported to affect protein localization [21,22,23], stability [24,25,26], and ion gating properties of the forming anion channel [27,28,29,30,31]. Likewise, stress responses upon proteasomal or endo-lysosomal dysfunction [24,32] and dysregulation of calcium homeostasis [33] are postulated to play a role in the pathogenesis of the bestrophinopathies. Bringing together the various lines of experiments to resolve the molecular pathologies of BEST1-associated phenotypes remains challenging.

Here, we utilized patient-derived human induced pluripotent stem cell (hiPSC) retinal pigment epithelium (RPE) (hiPSC-RPE) cell lines associated with phenotypic manifestations of BD, ADVIRC, or ARB to investigate functional consequences of *BEST1* mutations in an attempt to categorize the diverse types of mutations. We show that, depending on the respective mutation, hiPSC-RPE cells display clear differences in protein degradation pathways and ion gating processes. In view of these findings, we provide a first classification system of *BEST1* disease-associated mutations based on distinct molecular mechanisms resulting in the various phenotypic expressions of BEST1 pathologies.

## 2. Results

### 2.1. Assigning Best Vitelliform Macular Dystrophy (BD)-, Autosomal Dominant Vitreoretinochoroidopathy (ADVIRC)-, and Autosomal Recessive Bestrophinopathy (ARB)-Associated Mutations to the BEST1 Chicken Three-Dimensional (3D) Crystal Structure

**Figure 1:**
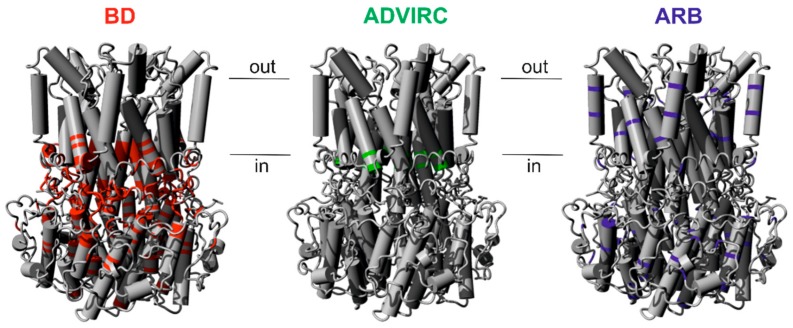
Assigning selected Best vitelliform macular dystrophy (BD)-, autosomal dominant vitreoretinochoroidopathy (ADVIRC)-, and autosomal recessive bestrophinopathy (ARB)-associated mutations to the chicken BEST1 structure. Crystal structure model of the homo-pentameric chicken BEST1 chloride channel [18]. Positions of BD- (red), ADVIRC- (green), and ARB- (blue) associated *BEST1* mutations are indicated. Genotype-specific mutations were selected according to defined criteria (see Materials and Methods) and are given in Supplementary Table S1.

Both autosomal dominant and autosomal recessive patterns of inheritance have been reported for pathologic *BEST1* mutations [9,34]. To explore a genotype-structure correlation we analyzed the spatial distribution of known disease-associated *BEST1* missense mutations and small in-frame deletions within the three-dimensional (3D) protein structure of chicken Best1. Based on criteria as defined in the method section, we selected 56 BD-, 16 ARB-, and the known five ADVIRC-associated mutations (Supplementary Table S1). For the autosomal dominant BD- and, in particular, the ADVIRC-associated mutations there is an apparent trend towards a location of the mutations around the neck and the structurally adjacent flexible helices [20] (Figure 1) implicating an important role of these amino acids in the ion gating processes. In contrast, most of the autosomal recessive mutations are located rather outside of this region (Figure 1).

### 2.2. In-Vitro Model of BD, ADVIRC, and ARB

To examine biological consequences of specific *BEST1* mutations in a cellular model that recapitulates native RPE structure, we developed patient-derived hiPSC-RPE lines initially derived from BD patients [22]. This offers the opportunity to investigate key molecular pathways in the pathogenesis of the bestrophinopathies. Here, we extend our previous analyses of mutant BEST1 protein to include additional BD but also ADVIRC and ARB mutant cell lines. Human iPSCs were derived from fibroblasts obtained from skin biopsies of 16 participants including three healthy controls (+/+ #1–#3), six BD patients (+/N11K, +/R218C, +/Q238R, +/A243V and two unrelated patients +/I295del #1 and +/I295del #2), two patients carrying ADVIRC mutations (+/V86M #1 and +/V86M #2), and two families segregating ARB (each family with one affected child carrying compound heterozygous BEST1 mutations N99K/R141H and A195V/L197PX26, respectively, and their clinically healthy parents +/N99K, +/R141H, +/A195V, and +/L197PX26, respectively) (Table 1 and patient information Supplementary Figure S1 and Table S2). Of note, three clones, +/+ #1, +/Q238R, and +/A243V, are established iPSC cell lines that were included in previous studies [16,22]. Furthermore, for 10 of the 16 cell lines, multiple independent hiPSC clones were used in the following analyses (Table 1). RPE differentiated from the hiPSCs displayed characteristic hexagonal morphology and pigmentation with typical high values of transepithelial electrical resistance (TER) after five weeks on Corning Transwell filter inserts (Supplementary Table S3). Only hiPSCs from ARB parent +/N99K produced low yields of hiPSC-RPEs showing low values of TER. This cell line was excluded from further analysis.

### 2.3. Localization, RNA, and Protein Expression of Mutant BEST1 in BD, ADVIRC, and ARB hiPSC-RPEs

**Figure 2:**
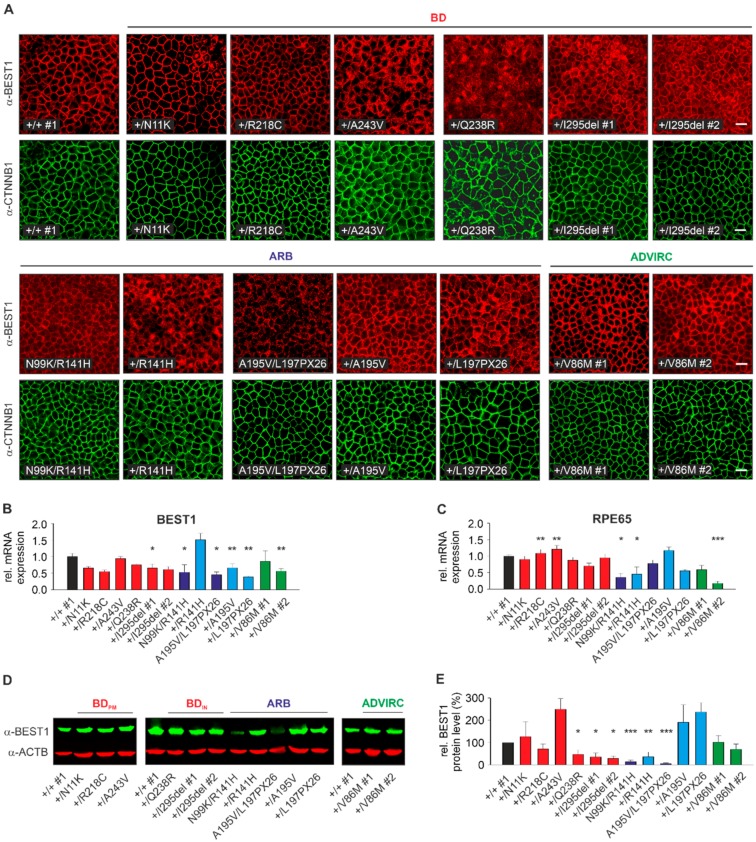
Localization, RNA, and protein expression analysis of normal and mutated BEST1. (**A**) Confocal immunofluorescence images of monolayers from control, BD, ARB, and ADVIRC hiPSC-RPEs after five weeks growth on Corning Transwell filter inserts using α-BEST1 and α-β-catenin antibodies. Scale bar: 20 µm. (**B***–***C**) Quantification of *BEST1* (**B**) and *RPE65* (**C**) expression by qRT-PCR of total mRNA extracted from BD, ARB, and ADVIRC hiPSC-RPEs relative to control (+/+ #1). Independent samples (*n* = 3) were measured in triplicates and normalized to *HPRT1*. Data were given as mean ± SD. Please note that Supplementary Figure S2 depicts individual variation of quantitative real-time reverse transcription polymerase chain reaction (qRT-PCR) measurements between individual control cell lines. (**D**) Representative Western blot images of whole cell lysates from control, BD, ARB, and ADVIRC hiPSC-RPEs using α-BEST1 antibody. Anti-β-actin was used as a loading control. (**E**) Quantification of BEST1 protein expression from (**D**) relative to control (+/+ #1) and normalized against β-actin from the same blot. Mean values are given as mean ± SD from two technical replicates per individual sample (n = 2–3). A summary of hiPSC clones used in the experiments is given in Supplementary Table S4. Statistical analyses were performed applying Kruskal-Wallis test, following post-hoc Dunn’s multiple comparisons test including Benjamini-Hochberg procedure: * = *p* < 0.05; ** = *p* < 0.01; *** = *p* < 0.001 and indicated relative to control (+/+ #1).

First, we examined the localization of BEST1 in normal, BD, ADVIRC, and ARB hiPSC-RPEs. Immunolabeling of BEST1 with human polyclonal antibody hBEST1-334 [22] in +/+ #1 confirmed co-localization with beta-catenin, a protein known to localize at the basolateral cell surface of polarized epithelial cells (Figure 2A). While a predominant basolateral plasma membrane (PM) staining similar to +/+ #1 was detected in three out of six BD-associated (+/N11K, +/R218C, +/A243V) and two ADVIRC cell lines, BD-associated cell lines +/I295del #1 and #2 and particularly +/Q238R demonstrated a large fraction of intracellularly retained mutant BEST1 protein. Furthermore, each of the hiPSC-RPE cell lines of the two ARB families exhibited a PM staining although with some degree of line-to-line variability, particularly for parent +/R141H. Fluorescence intensities were notably weaker in the two ARB patients compared to all other cell lines (Figure 2A).

To analyze *BEST1* mRNA expression, we next performed quantitative real-time reverse transcription polymerase chain reaction (qRT-PCR) analysis. Gene expression of *RPE65*, a specific marker for mature RPE, served as control. Each cell line strongly expressed *BEST1* and *RPE65* although there was some degree of variability in mRNA expression that is likely due to clonal effects (Figure 2B,C and Supplementary Figure S2). To further analyze the consequences of the premature stop codon of ARB mutation L197PX26 on *BEST1* mRNA expression we performed Sanger chain-terminating dideoxynucleotide sequencing to compare genomic and cDNA sequences from parent genotype +/L197PX26. Genomic sequencing identified overlapping sequencing traces of wildtype and mutant transcripts at heterozygous single nucleotide polymorphism rs1800007 in *BEST1* exon 2. In contrast, cDNA sequencing revealed exclusively the presence of a single transcript confirming nonsense-mediated mRNA decay (NMD) of mutant transcript L197PX26 whereas in ARB patient N99K/R141H both alleles of rs1800009 in exon 10 were present in the genomic and cDNA sample (Supplementary Figure S3A). In 2004, Yardley et al. showed that ADVIRC-associated mutations affect exonic splicing using an in-vitro minigene assay [8]. To analyze splicing effects of ADVIRC mutation *p*.(V86M) under more native conditions, PCR amplification of full-length BEST1 from hiPSC-RPE cDNA from control and the two ADVIRC patients revealed exclusively the correct amplicon of 1.7 kb indicating normal splicing of exon 4 (Supplementary Figure S3B). Sanger sequencing of the amplicon confirmed its homology to the correct *BEST1* transcript, thereby supporting previous findings [35].

Next, we quantified BEST1 protein expression in whole-cell lysates of hiPSC-RPEs from control, BD, ADVIRC, and ARB genotypes by Western blot analysis. We observed similar or even higher BEST1 expression relative to healthy control for BD mutants +/N11K, +/R218C, and +/A243V (127% ± 67%, 71% ± 23% and 249% ± 48%, respectively, hereinafter named “BD_PM_”), the two ADVIRC patients (102% ± 30% and 70% ± 24%) and for ARB parents +/A195V and +/L197PX26 (191% ± 79% and 237% ± 43%). For the latter, the rather high BEST1 protein expression was unexpected as anti-BEST1 antibodies target the C-terminal BEST1 protein. Since *BEST1* mRNA expression in +/L197PX26 is significantly lower than in ARB parent cell lines +/R141H and +/A195V, the results most likely reflect line-to-line variability in hiPSC-RPEs. In contrast, mislocalization of BEST1 protein in BD hiPSC-RPEs samples of +/Q238R, +/I295del #1 and #2 led to a significant reduction of BEST1 expression (47% ± 21%, 35% ± 18% and 29% ± 11%, respectively; hereinafter named “BD_IN_”). Unexpectedly, expression for ARB parent genotype +/R141H was rather weak (36% ± 21%) possibly due to clonal effects. BEST1 expression for the two ARB patients N99K/R141H and A195V/L197PX26 were dramatically reduced (14% ± 8% and 5% ± 3%) (Figure 2D,E).

### 2.4. Effect of BD-, ADVIRC-, and ARB-Associated Mutations on BEST1-Mediated Anion Permeability

**Figure 3:**
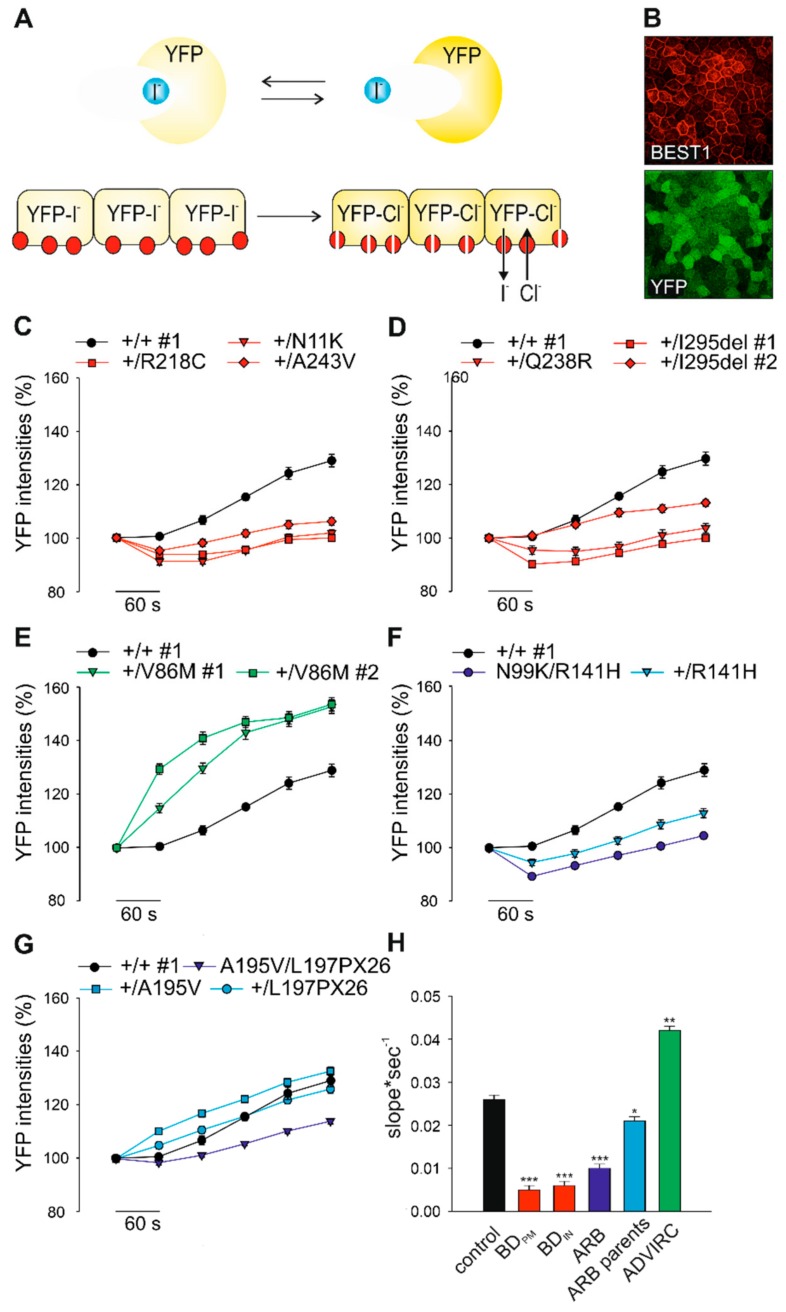
Consequences of genotype-specific *BEST1* mutations on anion permeability in hiPSC-RPEs. (**A**) Schematic illustration of the yellow fluorescence protein (YFP)-halide transport assay. Human iPSC-RPEs were transduced with YFP-expressing lentiviral particles. YFP fluorescence signals are quenched after addition of iodide ions (I^-^) and fully reversed after exchanging I^-^ with equimolar chloride ion (Cl^-^) solution. (**B**) Fluorescence micrograph of hiPSC-RPEs expressing YFP and normal BEST1 after immunostaining with an α-BEST1 antibody. Scale bar: 20 µm. (**C***–***G**) Kinetic of YFP fluorescence intensities over time in hiPSC-RPEs from representative control +/+ #1 and patients associated with BD_PM_ (**C**), BD_IN_ (**D**), ADVIRC (**E**), and the two ARB families (**F** and **G**). Recordings were taken at indicated time points after initial exposure to I^-^ followed by the addition of Cl^-^ containing solution. (**H**) Averaged bar graphs obtained from (**C***–***G**) showing recovery rates (slope*sec^-1^) from initial levels of YFP quenching to maximum fluorescence signals after 5 min of exposure to Cl^-^. Mean values are given as mean ± SE from six to 18 technical replicates per individual sample (*n* = 3–4). A summary of hiPSC clones used in the experiments is given in Supplementary Table S4. Statistical analysis was performed applying Kruskal-Wallis test, following post-hoc Dunn’s multiple comparisons test including Benjamini-Hochberg procedure: * = *p* < 0.05; ** = *p* < 0.01; *** = *p* < 0.001 and indicated relative to control.

To analyze BEST1-mediated anion transport in hiPSC-RPE cell lines we adapted an established halide transport assay [36] (Figure 3A). Human iPSC-RPEs were virally transduced with the yellow fluorescence protein (YFP)-based halide sensor YFP(H148Q/I152L) and seeded on black 96-well microtiter plates revealing a bright and uniform cell fluorescence highly sensitive to iodide ions (I^-^) (Figure 3B) [37]. Addition of I^-^ leads to specific YFP quenching over time and changes of fluorescence intensities can be monitored on a plate reader. After five weeks of growth on culture plates hiPSC-RPE cells were incubated with 100 mM I^-^ containing solution and quenching of YFP fluorescence signal intensities were measured after 6 min. Subsequently, after addition of the calcium ionophore A23187, I^-^ was replaced by equimolar chloride ions (Cl^-^). Anion permeability was then monitored by increasing YFP intensities as a result of BEST1-mediated I^-^ efflux in a time course of up to 5 min. In control cell lines +/+ #1–3 we observed a fast kinetic of increasing fluorescence intensities up to ~20% to 40% (Supplementary Figure S4). To demonstrate the utility of this assay for characterizing the function of BEST1, all hiPSC-RPE cell lines were analyzed under identical conditions. For the BD- and ARB-associated patient cell lines we found a significant decrease in fluorescence intensities when compared to control demonstrating strongly reduced channel activity (slope*sec^−1^: *p* < 0.001) (Figure 3C,D,F–H). ARB parent cell lines showed increased fluorescence signals compared to ARB patients demonstrating proper BEST1 channel function in the presence of a monoallelic autosomal recessive mutation (Figure 3F–H). Interestingly, hiPSC-RPEs of the two ADVIRC patients exhibited a ~60% slope increase of the fluorescence intensity rise compared to control (Figure 3E,H) suggesting a distinct effect of ADVIRC-related mutations on BEST1 channel function in comparison to BD- and ARB-associated mutations.

### 2.5. BEST1 Processing in BD and ARB hiPSC-RPEs via Distinct Degradation Pathways

Previously, we demonstrated high rates of protein turnover and distinct degradation mechanisms for autosomal dominant and recessive mutant BEST1 protein using a Madine-Darby canine kidney (MDCK) II in-vitro cell culture model stably expressing BD- and ARB-associated *BEST1* mutations [24]. Here, we investigated characteristics of the degradation pathways in the hiPSC-RPE model system of heterozygous BD-, ADVIRC-, and compound heterozygous ARB mutations.

**Figure 4:**
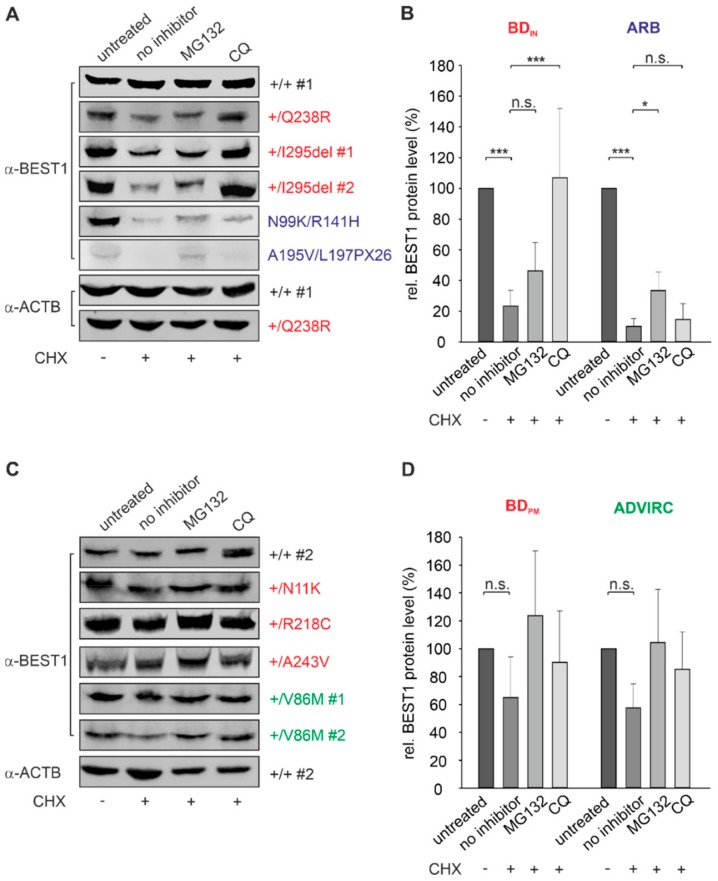
Degradation pathways for BEST1 clearance in BD, ARB, and ADVIRC hiPSC-RPEs. Representative Western blot images after α-BEST1 immunoblot staining of whole cell lysates from untreated and treated hiPSC-RPEs of (**A**) control +/+ #1 and patients associated with BD_IN_ and ARB, respectively. (**B**) Summary of BEST1 protein expression obtained from experiments shown in (**A**). Representative Western blot images after α-BEST1 immunoblot staining of whole cell lysates from untreated and treated hiPSC-RPEs of (**C**) control +/+ #2 and patients associated with BD_PM_ and ADVIRC, respectively. (**D**) Summary of BEST1 protein expression obtained from experiments shown in (**C**) relative to untreated samples and normalized against β-actin from the same blot. Samples in (**A**) and (**C**) were assayed after 12 h of CHX treatment (20 µg/mL) in the absence or presence of proteasomal inhibitor MG132 (40 µM) or lysosomal inhibitor chloroquine (CQ) (100 µM). In (**B**) and (**D**) graphs for BD_IN_, BD_PM_, ARB, and ADVIRC samples are averaged and mean values are given as mean ± SD from two technical replicates per individual sample (*n* = 13). A summary of hiPSC clones used in the experiments is given in Supplementary Table S4. Statistical analysis was performed applying Kruskal-Wallis test, following post-hoc Dunn’s multiple comparisons test including Benjamini-Hochberg procedure: * = *p* < 0.05; *** = *p* < 0.001; n.s. = not significant.

To analyze protein stability in the hiPSC-RPE cell lines we first quantified expression of remaining BEST1 protein after treating cells with cycloheximide (CHX), a known inhibitor of protein synthesis. After 12 h of CHX treatment protein quantity of BEST1 in hiPSC-RPEs +/+ #1 and #2 remained stable (Figure 4A,C), whereas the three BD_IN_ and the two ARB patient cell lines showed significant degradation relative to untreated cells (BD_IN_: 23% ± 10% and ARB: 10% ± 5%; *p* < 0.001) (Figure 4A,B). In contrast, localization of BEST1 at the cell surface of BD_PM_ and ADVIRC patient cell lines correlated with protection of BEST1 channel complexes from rapid degradation (Figure 4C,D).

To further investigate the underlying degradation pathways of unstable BEST1 in BD_IN_ and ARB cell lines, we used MG132 and the weak base chloroquine (CQ) to selectively inhibit the proteasomal and endo-lysosomal degradation pathway, respectively. Our results demonstrate that degradation of BEST1 protein in the two ARB patient cell lines is significantly inhibited only by the proteasomal inhibitor MG132 (*p* < 0.01) (Figure 4A,B). In contrast, MG132 failed to reveal an effect in BD_IN_ hiPSC-RPEs, while protein expression level increased significantly after incubation with the lysosomal inhibitor chloroquine; the latter suggesting that mutated autosomal dominant BEST1 protein undergoes degradation via the endo-lysosomal pathway (Figure 4A,B). Together, our results suggest distinct cellular mechanisms for the degradation of unstable BEST1 in BD and ARB patient cell lines, well in agreement with previous results [24,38].

### 2.6. Altered Lysosomal Homeostasis in BD hiPSC-RPEs

**Figure 5:**
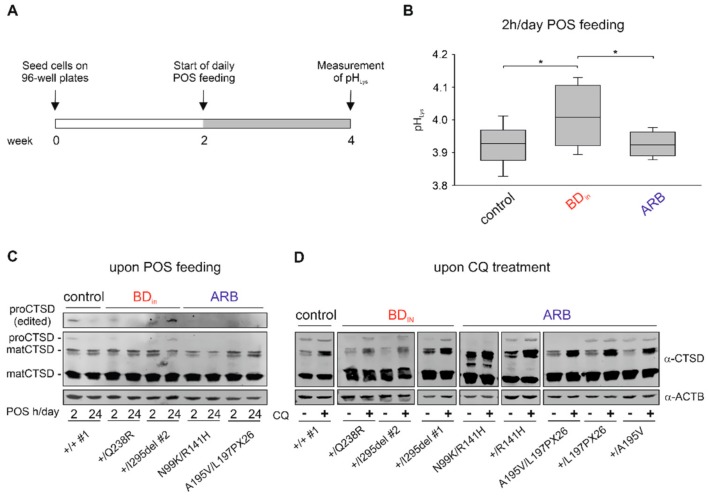
Effect of *BEST1* mutations on lysosomal homeostasis in BD_IN_ and ARB hiPSC-RPEs. (**A**) Schematic protocol used to assess lysosomal pH (pH_Lys_) after daily photoreceptor outer segment (POS) feeding. (**B**) Box plot showing pH_Lys_ in hiPSC-RPEs from +/+ #1 and +/+ #2 (control), +/Q238R, and +/I295del #2 (BD_IN_) and the two ARB patients after two weeks of POS feeding (~20 POS/cell for 2 h/day). Ratios of fluorescence excited at 385 nm and 330 nm were determined through fluorescence measurements on a plate reader using the ratiometric indicator dye LysoSensor^TM^ Yellow/Blue DND-160. Absolute values of pH_Lys_ were calculated relative to a standard curve for each cell line. Mean values are given as mean ± SD from five technical replicates per individual sample (*n* = 2). Statistical analysis was performed applying Kruskal-Wallis test, following post-hoc Dunn’s multiple comparisons test including Benjamini-Hochberg procedure: * = *p* < 0.05. (**C** and **D**) Analysis of proteolytic enzyme cathepsin D (CTSD) maturation. Representative Western blot images of whole cell lysates from BD_IN_ and ARB hiPSC-RPEs (**C**) after one week of daily POS feeding for 2 h or 24 h and (**D**) before (−) and after (+) treatment with 100 µM CQ for 12 h. Antibody against CTSD was used to detect two mature (matCTSD; ~34 kDa and ~14k Da) and a short-lived form (proCTSD; ~55 kDa) of CTSD. Please note that proCTSD (edited) in (**C**) represents an overexposed image to demonstrate absence of the 55 kDa band in the ARB samples. A summary of hiPSC clones used in the experiments is given in Supplementary Table S4.

We next analyzed whether enhanced degradation of BD_IN_-associated BEST1 in the lysosome may affect lysosomal pH (pH_Lys_) in BD hiPSC-RPEs. Measurement of pH_Lys_ was performed with hiPSC-RPEs of BD_IN_ patient genotypes +/Q238R and +/I295del #2, the two ARB patients, and controls +/+ #1 and +/+ #2 using the ratiometric indicator dye LysoSensor^TM^ Yellow-Blue DND-160 [39,40,41]. After parallel cultivation of cell lines in black 96-well microtiter plates for two weeks no difference in pH_Lys_ was seen between control and BD_IN_ hiPSC-RPE cells (control: 4.25 ± 0.02, BD_IN_: 4.23 ± 0.03) (Supplementary Figure S5). However, when challenged with chronic daily shed photoreceptor outer segment (POS) feeding for two weeks (Figure 5A), we found a small but significant elevation of pH only in the lysosomes of BD_IN_-associated hiPSC-RPEs whereas measurements in the two ARB cell lines were comparable to controls (control: 3.93 ± 0.06, BD_IN_: 4.01 ± 0.01, ARB: 3.92 ± 0.04; *p* > 0.05).

As variability of pH_Lys_ can influence the activity of lysosomal enzymes [41], we further examined the maturation of the proteolytic enzyme cathepsin D (CTSD), representing a major lysosomal protease controlling lysosomal function [42]. CTSD is synthesized in the ER as an inactive and short-lived pro-protein (proCTSD; ~55 kDa) and is processed after several proteolytic steps at acidic pH to a mature and active protease (matCTSD) composed of a ~34 kDa heavy chain and a smaller ~14 kDa light chain [43]. Western blot analysis was performed with protein lysates from hiPSC-RPEs of BD_IN_, ARB patients and parent samples, as well as healthy controls after inducing lysosomal stress by POS feeding as a natural stressor (Figure 5C) or the application of 100 µM chloroquine (Figure 5D) to elevate lysosomal pH. Under both experimental conditions, we found solely the two 34 and 14 kDa mature forms of CTSD in hiPSC-RPE lysates from ARB patients while the 55 kDa proenzyme was not detectable (Figure 5C,D). In contrast, proCTSD is present after chloroquine treatment in BD_IN_-associated hiPSC-RPEs while untreated samples remained unaffected. One week of daily POS feeding confirmed the presence of a ~55 kDa protein only in hiPSC-RPEs harboring dominant BEST1 mutations. Of note, the immature proenzyme form is also detected in hiPSC-RPEs from healthy control and unaffected parents indicating that degradation of native BEST1 through homeostatic regulation of membrane protein turnover is also targeted to the lysosome as described for many PM proteins [44,45]. Together, these results indicate that degradation of BEST1 affects the integrity of lysosomes in hiPSC-RPEs of BD_IN_ but not ARB patients.

### 2.7. Classification of BEST1 Mutations

**Figure 6:**
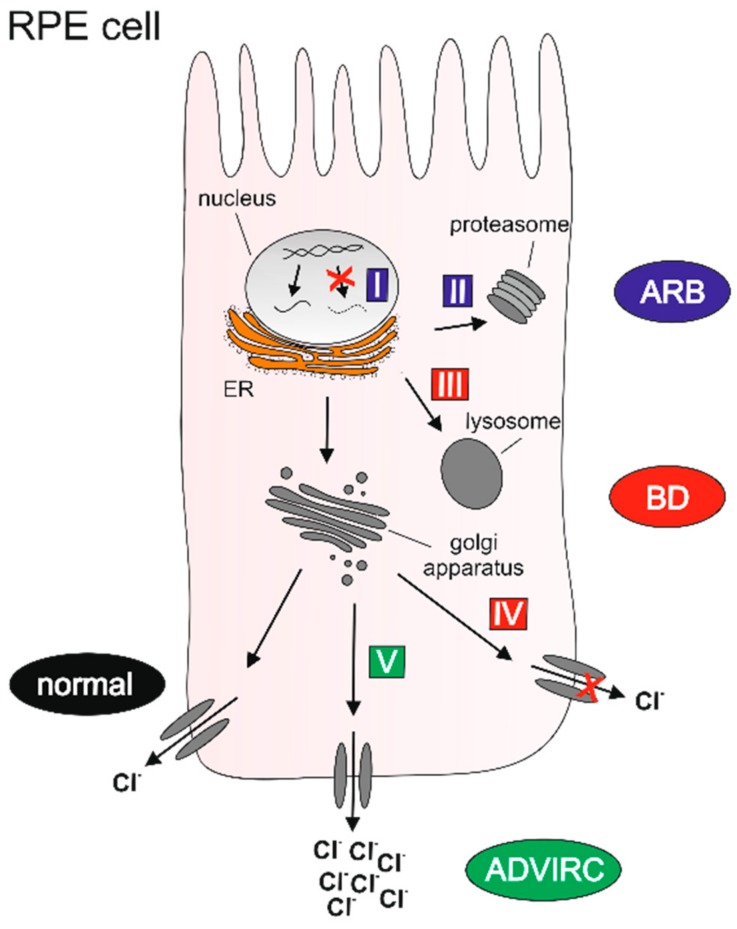
Graphical classification of distinct molecular mechanisms underlying genotype-specific *BEST1* mutations. Class I: ARB-associated defect of BEST1 protein synthesis; class II: ARB-associated ER-related proteasomal degradation; class III: BD_IN_-associated post-ER-related lysosomal degradation; class IV: BD_PM_-associated reduced ion gating, and class V: ADVIRC-associated enhanced ion gating. For further details on the classification of BEST1 mutations, please see Table 2. (Drawing modified from Figure 3 in DOI: 10.1055/a-1065-2129. Permission for use was granted by the Thieme Group, Stuttgart, Germany.).

Based on our results on the molecular mechanisms of pathologic BEST1 variants we classified the mutations causing the distinct bestrophinopathies into five different classes (I–V) (Table 2). The classes distinguish mutations that lead to defects in BEST1 synthesis, channel function, or protein structure that—depending on the type of mutation—activate different degradation pathways. BEST1 protein is either not synthesized (class I), processed by the (ER)-related proteasomal (class II), or endo-lysosomal (class III) degradation pathway or shows reduced (class IV) or enhanced ion transport (class V), respectively (Figure 6). Class I and II mutations exclusively follow an autosomal recessive mode of inheritance. Monoallelic carriers of these two classes generally show no ocular symptoms. In contrast, heterozygous carriers of class III, IV, and V mutations exert a dominant-negative effect on the functionality of the channel due to their contribution of defective BEST1 subunits to the final channel complex formation [18,19,20].

## 3. Discussion

Patient-derived hiPSC-RPE cells are a valuable tool to explore the pathophysiology of degenerative retinal diseases in a cell model closely resembling the native cell type of primary pathology [16]. It can also be used to evaluate potential therapies. Here, we investigated a large number of patient-derived hiPSC-RPEs harboring pathologic *BEST1* mutations associated with three distinct phenotypes within the group of bestrophinopathies. The aim of the study was to further add to our knowledge about the molecular pathologies underlying the clinically distinguishable phenotypes. Our results demonstrate that hiPSC-RPEs derived from BD, ADVIRC, and ARB patients display specific properties in BEST1 expression, localization, degradation pathway, and ion gating processes.

Specifically, we show that autosomal dominant *BEST1* mutations in BD and ADVIRC hiPSC-RPEs escape ER-associated degradation and instead are recognized by a post-ER quality control probably at the Golgi complex (BD_IN_) or on the cell surface (BD_PM_ and ADVIRC) disassembling the mutant protein complexes in the lysosome. We found that anion transport function in hiPSC-RPEs unequivocally discriminates between dominant BD and ADVIRC mutations as anion permeability is considerably higher in ADVIRC hiPSC-RPEs cells when compared to controls. BD hiPSC-RPEs showed significantly reduced ion transport function. Our results also show that ARB-associated frameshift mutation L197PX26 is recognized by the eukaryotic mRNA NMD control mechanism likely resulting in loss-of-function of mutated BEST1. In contrast, the presence of mRNA transcripts in hiPSC-RPEs from patients carrying ARB-associated missense mutations seems to result in the formation of greatly unstable mutant subunits that are recognized by the ER control machinery and thereby are prone for degradation via the proteasome.

For the first time, this study provides data on the effect of ADVIRC-associated mutation BEST1-V86M on BEST1 localization, protein expression and anion transport function. While localization of mutated BEST1 to the basolateral plasma membrane was established in our study in two independent patient cell lines well in agreement with results from overexpression studies in a MDCKII cell model [18], ADVIRC-associated mutation (c.704T>C; p.(V235A)) was previously reported to be mislocalized at least in part to the apical surface of hiPSC-RPEs from an ADVIRC patient [35]. These contradictory results could suggest that proper BEST1 localization or a failure to correctly traffic to the cell membrane is rather mutation-dependent than an ADVIRC-specific characteristic and further underscores the necessity to model additional ADVIRC mutations on the basis of the hiPSC-RPE cell culture model. Furthermore, studies have shown that ADVIRC mutations p.(V86M), p.(Y236C), p.(V239M), and p.(V235A) affect BEST1 mRNA splicing using a minigene assay in HEK293 cells [8,46] while ADVIRC mutation p.(G83D) failed to exhibit any effect on mRNA splicing [47]. In the present study, we found no evidence for alternative splicing in the ADVIRC-associated hiPSC-RPE cell lines well in agreement with earlier findings from Carter et al. [35]. A major finding of the latter study is that ADVIRC-associated mutation p.(V86M) significantly increased BEST1-associated anion transport in comparison to control cell lines thereby supporting evidence from earlier observations [28]. Electrophysiological patch-clamp analysis of HEK293 cells expressing ADVIRC-associated mutation p.(Y236C) revealed a significantly enlarged Cl^-^ current when compared to normal BEST1 protein [30]. The observed increase in Cl^-^ conductance might be attributed to an enhanced maximal open probability as was similarly shown for other diseases [48]. Findings from a recent study support this conclusion as 3D structures from single-particle cryo-EM analysis failed to reveal a closed gate conformation for ADVIRC-associated *BEST1* mutation p.(Y236C) and for mutation p.(W287F) indicating that an open state is preferential for these two *BEST1* mutations [21]. Hence, a continuous chloride transport across the cell membrane in the early development of the human eye may account for the distinct ocular phenotype and possibly the developmental anomalies in ADVIRC patients.

The RPE is a highly active phagocytic cell complex and loaded with a substantial number of lysosomes to accomplish the high load of daily POS clearance [49]. Incomplete degradation of internalized outer segments is thought to accumulate as lipofuscin in the lysosomal compartment of the RPE gradually contributing to a decline in cell function [50]. Since the accumulation of lipofuscin-like material is a characteristic feature of the BD phenotype, it appears coherent to analyze whether mutant BEST1 could affect lysosomal function. Up to now there has been no direct link between POS upload and lysosomal dysfunction in BEST1-related disease. Here, we show that upon daily POS feeding there is an association with an increasing pH in acidic lysosomes of two independent BD_IN_-associated hiPSC-RPE cell lines. This is in contrast to measurement in ARB and control cell lines where pH values remained at steady state despite POS feeding. Our findings are further supported by demonstrating that an elevation of lysosomal pH in BD_IN_ upon daily POS feeding or CQ treatment promotes incomplete processing and maturation of lysosomal catalytic enzymes. Hence, continuous degradation of autosomal dominant BD_IN_ and likely BD_PM_ protein in the lysosomal machinery could be the starting point for BD pathology while negatively influencing the overall degradation capacity of RPE lysosomes.

In contrast, continuous degradation of autosomal recessive ARB protein in the ER is likely part of manifestations of ARB pathology by exhausting chaperone activity over time and disturbing ER homeostasis. Only recently, we have shown in a MDCKII cell model stably expressing ARB-associated BEST1 mutations, that proteasome-associated degradation of mutant autosomal recessive BEST1 increases susceptibility for ER stress by analyzing the mRNA expression of X-box binding protein 1 (XBP1) in response to ER stress [24]. Contrary to the previous findings, we show in the present study that continuous degradation of misfolded/unfolded mutant BEST1 protein in the ER of ARB-hiPSC-RPEs triggers the unfolded protein response, a condition known to reflect ER stress. Importantly, we here demonstrate that unstable BEST1 protein in the two ARB-associated hiPSC-RPE cell lines is degraded via the ER-associated proteasomal machinery which distinguishes the autosomal recessive from the autosomal dominant phenotype. Future experiments will be needed to further delineate the impact of ARB-related mutations in hiPSC-RPEs on ER homeostasis.

Our BEST1 mutation classification scheme is based on genetic and molecular data and raises the question whether the different classes correlate with specific phenotypic features, such as clinical findings or electrophysiological measurements. Where EOG data have been available in our study, ARB-, BD-, and ADVIRC patients reveal a reduced Arden ratio (<1.8), while heterozygous ARB parents showed a normal coefficient as defined by >2.0 (see also Supplementary Table S2). As the Arden ratio provides a dichotomic non-quantifiable trait, it is not useful to differentiate the effect of defined BEST1 mutations. Similarly, a correlation of BEST1 mutations to specific clinical phenotypic features is challenging and may require large sample sizes of patients with identical BEST1 mutations, however, in this study we only included one patient per mutation in the in vitro analysis.

The disparate effects of disease-associated *BEST1* mutations on molecular characteristics and BEST1 function, as shown in this study, calls for conscious and deliberate views when targeted treatment options are considered in BEST1-associated disease. While there is currently no effective therapy for any of the three bestrophinopathies, first promising results of a *BEST1* gene replacement therapy have been reported for a canine model of ARB [51]. The introduction of a normal *BEST1* gene copy into the eyes of homozygous or compound heterozygous animals appears to improve ocular symptoms. Nevertheless, thorough functional examination of each autosomal recessive BEST1 mutation is mandatory to determine the appropriate approach for the gene replacement therapy. Only recently, inconclusive results on protein degradation routes have been reported for ARB-associated missense mutations D312N and V317M [38]. This, however, may be greatly influenced by specific experimental settings and cell lines used. Of note, our results suggest that only ARB patients with homozygous or compound heterozygous class I mutations will have the most benefit as only the complete loss of BEST1 protein can be fully compensated for by an addition of functional gene copies. In contrast, together with findings of our previous study [24], we expect that ARB patients with at least one class II missense mutation should continue to produce mutant protein thereby inducing ER stress over time [24] and consequently gradually impairing the degradation capacity of the proteasomal machinery [52]. Although these patients may experience an initial improvement of their condition, in the long-term progression of the degenerative disease with corresponding functional limitations would be expected as multifocal subretinal deposits and abnormal autofluorescence in the ARB phenotype may further develop from ER stress-induced accumulation of cytosolic aggregation-prone proteins. Based on our model, it may be more appropriate for these patients to follow a so-called “ablate-and-replace” strategy for full recovery of vision as suggested by Tsai et al. [53].

In principle, gene replacement therapies could also improve the symptoms for the autosomal dominant forms of the bestrophinopathies. With a continuous production of normal BEST1 protein, the ratio of mutant and normal BEST1 subunit available for homo-pentameric channel assembly should shift in favor of the normal subunits. Nevertheless, based on our data, we hypothesize that the consequences associated with a continuous degradation of mutated BEST1 in the RPE lysosomes would probably jeopardize a long-term therapeutic success. Complications should mainly stem from lysosomal stress-induced accumulation of shed POS due to an increase in lysosomal pH. However, aiming at a removal of the mutant *BEST1* gene copy could be a meaningful and efficient therapeutic approach, as the intact allelic gene copy may be sufficient to restore chloride conductance in the RPE. Utilizing the specificity of the CRISPR/Cas9 genome editing system, allele-specific targeting of single point mutations associated with various autosomal dominant diseases have already been successfully tested in some cell systems [54,55] and animal models [56,57].

Taken together, this study provides a first comprehensive analysis of the cellular and molecular mechanisms underlying the distinct pathologies of the three classical forms of the bestrophinopathies. Only with the full understanding of the cellular effects on the pathologies of the respective disease entity a targeted and efficient therapeutic approach is plausible and promising to be successful in the long-term.

## 4. Materials and Methods

### 3D Mutation Modelling of BEST1 Variants

For in-silico modelling, the 3D structure of chicken Best1 (cBest: PDB ID 4rdg) [15] was implemented in the software YASARA Structure (Version 18.2.7, YASARA Biosciences GmbH, Austria). Autosomal dominant and recessive genetic variants depicted in the model were selected from a list of 1315 *BEST1* mutations (i) that have been published between 1997 and March 2018 or (ii) from our patient cohort (Institute of Human Genetics of Wuerzburg (1997–2005) and Regensburg (2006–2018)). Mutations were categorized as autosomal dominant or autosomal recessive according to the following criteria: (I) An unambiguous autosomal dominant or autosomal recessive inheritance has been reported for at least three independent patients; (II) a biallelic inheritance has never been reported for an autosomal dominant mutation; or (III) functional characterization of a mutation was reported. Mutations that have been found exclusively in a homozygous condition were excluded.

### Generation of Human Induced Pluripotent Stem Cells (hiPSCs) and Differentiation into Retinal Pigment Epithelium (RPE) Cells

Isolation of adult human dermal fibroblast, generation of hiPSCs, and differentiation to RPE cells was performed as previously described with minor changes in the reprogramming process [3]. Briefly, hiPSCs were generated after electroporation of fibroblasts with episomal plasmids pCBX-EBNA, pCE-hSK, pCE-hUL, pCE-hOCT3/4, and pCE-mp53DD according to Okita et al. [58] (all plasmids were kind gifts from Shinya Yamanaka; plasmids #41857, #41814, #41855, #41813, and #41856 were purchased from Addgene, Massachusetts, USA) using the Amaxa Nucleofector Electroporation device (program U-23) and the Human Dermal Fibroblast NucleofectorTM Kit VPD-1001 (Lonza, Basel, Switzerland). For further experiments, hiPSC-RPE cells were cultured for five weeks on six-well Corning Transwell filter inserts (0.4 µm pore size, 657641, Greiner, Kremsmünster, Austria) or black 96-well microtiter plates coated with Matrigel Growth Factor Reduced (GFR) Basement Membrane Matrix (dilution 1:30, 356252, Corning, New York, USA).

### Measurement of Transepithelial Resistance (TER)

TER of hiPSC-RPEs grown on six-well Corning Transwell filter inserts was measured using an epithelial volt/ohm meter (EVOMX) (World Precision Instruments, Berlin, Germany). A detailed procedure was described previously [5].

### Antibodies

Primary antibody rabbit polyclonal hBEST1-334 was described previously [22] and used in 1:250 (immunocytochemistry, ICC) or 1:2500 (Western blot, WB) dilution. Monoclonal mouse antibodies β-catenin (ICC dilution 1:1000, 610153, BD Biosciences, Franklin Lakes, USA), β-actin (WB dilution 1:10 000, #A5441, Sigma-Aldrich, St. Louis, USA), and rabbit monoclonal antibody cathepsin D (WB dilution 1:500, #ab75852, Abcam, Cambridge, UK) were commercially available. Western blot experiments were performed with secondary antibodies goat near-infrared fluorescent dyes (IRDye 1:10,000) (Lycor, Bad Homburg, Germany). Secondary antibodies for immunofluorescence were goat Alexa 594- conjugated anti-rabbit and Alexa 488-conjugated anti-mouse (dilution 1:1000, Thermofisher Scientific, Waltham, USA).

### Immunofluorescence

Monolayers of hiPSC-RPEs grown on Corning Transwell filter inserts were fixed with 4% PFA for 10 min at room temperature (RT). Cells were washed three times with PBS and blocking solution (10% normal goat serum, 0.3% Triton X-100 in PBS) was added for 25 min at RT. Primary and secondary antibodies were incubated over night at 4 °C, respectively. Cells were embedded in Dako Fluorescence Mounting Medium (#S3023, Agilent Technologies, Santa Clara, USA) and imaged using confocal microscope LSM510 and Zeiss LSM Image Browser software (Version 4.0.0.157, Zeiss, Oberkochen, Germany).

### RNA Isolation, Reverse Transcription (RT) and Real-Time Quantitative Reverse Transcriptase-Polymerase Chain Reaction (PCR) (qRT-PCR)

Total RNA was isolated from hiPSC-RPEs using the RNeasy Mini Kit (Qiagen, Hilden, Germany) according to the manufacturer’s instructions including an on-column DNase digestion (Qiagen, Hilden, Germany). First-strand cDNAs from 1 µg of total RNA were synthesized using the RevertAid^TM^ Reverse Transcriptase and Random Hexamer Primers (Thermo Fisher Scientific, Waltham, USA). For qRT-PCR analysis, oligonucleotide primers were designed using Roche Library Probes (Roche, Basel, Switzerland) (Supplementary Table S5). The reaction was performed with 2x TaqMan Gene Expression Master Mix and run on a QuantStudio^TM^ 5 Real-Time PCR System (Life Technologies, Carlsbad, USA). Data were analyzed following the ΔΔCt method.

### DNA Extraction, PCR Amplification and Sanger Sequencing

Genomic DNA (gDNA) was extracted from hiPSC-RPEs according to Lairds et al. [59]. GoTaq^®^ DNA Polymerase (#M317, Promega, Mannheim, Germany) was used for PCR amplification of gDNA and cDNA. For Sanger sequencing the BigDye Terminator v1.1, v3.1 Cycle Sequencing Kit (Life Technologies, Carlsbad, USA) was utilized and reactions run on an Abi3130X1 Genetic Analyzer (Applied Biosystems, Thermo Scientific, Waltham, USA). Oligonucleotide primer sequences are given in Supplementary Table S6.

### Cycloheximide (CHX) Treatment 

Cycloheximide (CHX) (#C4859, Sigma-Aldrich, St. Louis, USA), chloroquine phosphate (CQ) (#LKT-C2950.25, Biomol, Hamburg, Germany), and MG132 (#Cay-10012628, Cayman Chemical, Ann Arbor, USA) were obtained commercially. MG132 was available as a stock solution of 40 mM in dimethyl sulfoxide (DMSO). Human iPSC-RPEs were treated with dilutions of CHX (20 µg/mL) in the presence or absence of 40 µM MG132 or 100 µM CQ for 12 h at 37 °C.

### Protein Sample Preparation, Sodium Dodecyl Sulfate (SDS) Page and Quantitative Western Blot Analysis 

Whole cell lysates were prepared by homogenization in 1 x PBS. Sodium dodecyl sulfate (SDS) sample buffer was added and protein extracts were sheared on ice with fifteen 1-sec pulses at 30% amplitude using a Vibra-Cell sonicator (Sonics, Newtown, USA). Samples were then separated on SDS-polyacrylamide gel electrophoresis with 10% (for BEST1) or 12.5% (for CTSD) gels and subsequently transferred onto Immobilon-FL PVDF membrane (Milipore, Bedford, USA). Primary and secondary antibodies were incubated over night at 4 °C, respectively. Visualization of the fluorescent labeled protein was carried out using the Odyssey FC Imager (LI-COR Biosciences, Lincoln, USA). Signal intensities were quantified with the Image Studio software (Version 4.0, LI-COR Biosciences, Lincoln, USA) and normalized against β-Actin signals from the same blot. 

### Generation of Yellow Fluorescence Protein (YFP) Lentiviral Vector and Lentiviral Transduction

The YFP(H148Q/I152L) plasmid was kindly provided by Luis J.V. Galietta [36]. Full-length YFP(H148Q/I152L) was generated by PCR amplification using primer pair YFP-AgeI-F (5′-acc ggt acc atg gtg agc aag ggc gag ga-3′)/YFP-EcoRI-R (5′- gaa ttc tta ctt gta cag ctc gtc ca -3′) and subcloned into the pLJM1-EGFP vector (plasmid #19319 was a generous gift from David Sabatini [60] and purchased from Addgene, Massachusetts, USA). Human iPSC-RPE cells were transduced by lentiviral particles after co-transfecting HEK293T cells with YFP(H148Q/I152L) and helper plasmids pMD2.G and psPAX2 (plasmids #12259 and #12260 were kind gifts from Didier Trono and purchased from Addgene, Massachusetts, USA) using the calcium phosphate transfection method. After 72 h cells were cultured in selection medium containing 3 µg/mL Puromycin Dihydrochloride (#A1113802, Thermo Fisher Scientific, Waltham, USA).

### YFP-Halide Transport Assay

Human iPSC-RPE cells expressing YFP(H148Q/I152L) were cultured for five weeks on black 96-well microtiter plates. Cells were incubated for 6 min with I^-^ containing solution (100 mM NaI, 5 mM KCl, 1 mM MgCl_2_, 2 mM CaCl_2_, 10 mM HEPES, 5 mM D-glucose, mannitol is added for osmolality of 300 mosmol/kg) in the presence of the calcium Ionophore A23187 (5 µM; #C7522, Sigma-Aldrich, St. Louis, USA) and quenched YFP fluorescence signals were measured in a FLUOstar OPTIMA Microplate Reader (BMG Labtech, Allmendgruen, Germany). Subsequently, I^-^ was replaced with equimolar Cl^-^ containing solution (100 mM NaCl, 5 mM KCl, 1 mM MgCl_2_, 2 mM CaCl_2_, 10 mM HEPES, 5 mM D-glucose, mannitol is added for osmolality of 300 mosmol/kg) in the presence of calcium Ionophore A23187. YFP fluorescence intensities were monitored in 60 sec intervals in a time course of 5 min.

### Isolation and Feeding of Photoreceptor Outer Segments (POS) 

Purification of photoreceptor outer segments (POS) from porcine retinae was performed via ultracentrifugation following a protocol previously described [5]. For experiments with daily POS feeding, POS aliquots were freshly diluted in Opti-MEM^TM^ media (Thermo Fisher Scientific, Waltham, USA) and sonicated in an ultrasonic water bath at a frequency of 10% for 5 min. Afterwards, POS were passed through a 5 µm cell strainer (PluriSelect Life Science, Leipzig, Germany) and resuspended in fresh RPE media. Human iPSC-RPE cells on six-well Corning Transwell filter inserts or black 96-well microtiter plates were incubated for 2 or 24 h with an equivalent amount of ~20 POS/cell. Subsequently, hiPSC-RPEs were vigorously washed with KnockOut^TM^ DMEM (Thermo Fisher Scientific, Waltham, USA) to remove remaining POS from the cell surface before adding fresh RPE media.

### Lysosomal pH Measurement

Lysosomal pH was measured using the commercially available dye LysoSensor^TM^ Yellow-Blue DND-160 (Thermo Fisher Scientific, Waltham, USA) according to the manufacturer’s instructions. In brief, cells were incubated with 1 µM LysoSensor^TM^ solved in isotonic solution (20 mM MES, 5 mM KCl, 120 mM NaCl, 1 mM MgCl_2_, 2 mM CaCl_2_, 10 mM HEPES, 10 mM Glucose, pH 7.4) for 5 min and fluorescence intensities were measured at 330 nm and 385 nm on a TECAN Spark Microplate Reader (Tecan Group, Maennedorf, Switzerland). A calibration curve was established for every individual cell line grown on a black 96-well microtiter plate by incubation of cells for 15 min with isotonic solutions with defined pH values (pH 3.0–5.0). Nigericin (10 µM) and Monensin (10 µM) sodium salt (Sigma-Aldrich, St. Louis, USA) were added to dissipate intracellular pH gradients.

### Statistical Analysis

Data were expressed as mean ± standard deviation (SD), YFP fluorescence intensities and slopes were expressed as mean ± standard error (SE). Normality testing was conducted by Shapiro-Wilks test. Statistical analysis was performed applying Kruskal-Wallis test for non-normal data, following the post-hoc Dunn’s multiple comparisons test including Benjamini-Hochberg procedure. Significance was reported for * = *p* < 0.05; ** = *p* < 0.01; *** = *p* < 0.001.

## Figures and Tables

**Table 1:** hiPSC-RPE cell lines used in this study.

Amino Acid Exchange	Nucleotide Exchange	Alias (Clone Number)	Gender	Clinical Findings
				
+/+ #1	-	MK (#22, #27b)	m	reference
+/+ #2	-	AM (#13, #260)	f	reference
+/+ #3	-	NG (#1)	f	reference
+/N11K *	33T>G	MW (#232)	m	BD
+/R218C *	652C>T	MO (#214)	m	BD
+/A243V	728C>T	SK (#16)	m	BD
+/Q238R	713A>G	DK (#Pka, #Pkb)	m	BD
+/I295del #1 *	884_886delTCA	AP (#187)	m	BD
+/I295del #2 *	884_886delTCA	MD (#18)	m	BD
N99K/R141H *	422G>A/297C>A	LA (#29, #33)	f	ARB
+/N99K	422G>A	EA (-)	f	ARB parent (healthy)
+/R141H *	297C>A	MA (#74, #77)	m	ARB parent (healthy)
A195V/L197PX26 *	584C>T/590_615del26	TT (#178, #180)	m	ARB
+/A195V *	584C>T	PT (#172, #176)	m	ARB parent (healthy)
+/L197PX26 *	590_615del26	AT (#166, #168)	f	ARB parent (healthy)
+/V86M #1 *	256G>A	IS (#53a, #53b)	f	ADVIRC
+/V86M #2 *	256G>A	JS (#52a, #52b)	m	ADVIRC

* see clinical information in Supplementary Figure S1 and Table S2.

**Table 2:** Classification of BEST1 mutations.

Class I	Premature termination stop codons No detectable BEST1 protein Homozygous or compound heterozygous conditions lead to ARB (e.g., p.(L197PX26))
Class II	Synthesis of unstable BEST1 protein Recognized by the ER quality control and degraded via the proteasome Homozygous or compound heterozygous conditions lead to ARB (e.g., p.(R141H) and p.(A195V))
Class III	Synthesis of unstable BEST1 protein although not recognized by the ER quality control Mutant BEST1 subunits incorporate into the pentameric channel complex and exert a dominant-negative effect Unstable channel complexes are mislocalized to intracellular compartments and are recognized by the endo-lysosomal quality control for degradation in the lysosome Monoallelic mutations lead to autosomal dominant BD (e.g., p.(Q238R))
Class IV	BEST1 is localized to the plasma membrane BEST1 mutation reveals a negative effect on BEST1 channel open conformation severely limiting anion permeability Monoallelic mutations lead to autosomal dominant BD (e.g., p.(R218C)).
Class V	BEST1 is localized to the plasma membrane BEST1 mutation reveals a negative effect on BEST1 channel closed conformation severely increasing anion permeability Monoallelic mutations lead to autosomal dominant ADVIRC (p.(G83D), p.(V86M), p.(V235A), p.(Y236C) and p.(V239M))

## Supplementary Materials

Supplementary materials can be found at https://www.mdpi.com/1422-0067/21/5/1597/s1.
