# Supplementary material for: Mutation-Dependent Pathomechanisms Determine the Phenotype in the Bestrophinopathies
Source: Int J Mol Sci. 2020 Feb 26;21(5):1597. doi: 10.3390/ijms21051597 (PMC7084480; doi:10.3390/ijms21051597)
Supplement: Supplementary file 1 [file ijms-21-01597-s001.pdf]

Mutation-dependent pathomechanisms determine the phenotype in the bestrophinopathies

Anna-Lena Nachtigal[1*], Andrea Milenkovic[1*], Caroline Brandl[1,2,3], Heidi L. Schulz[1], Lisa M. J. Duerr[1], Gabriele E. Lang[4], Charlotte Reiff[5], Philipp Herrmann[6], Ulrich Kellner[7], Bernhard H.F. Weber[1]

Supplementary Information

This file contains Supplementary Figures S1 to S5 (including Supplementary Figure Legends) and Supplementary Tables S1 to S6 (including Supplementary Table headings).

Supplementary Figure S1

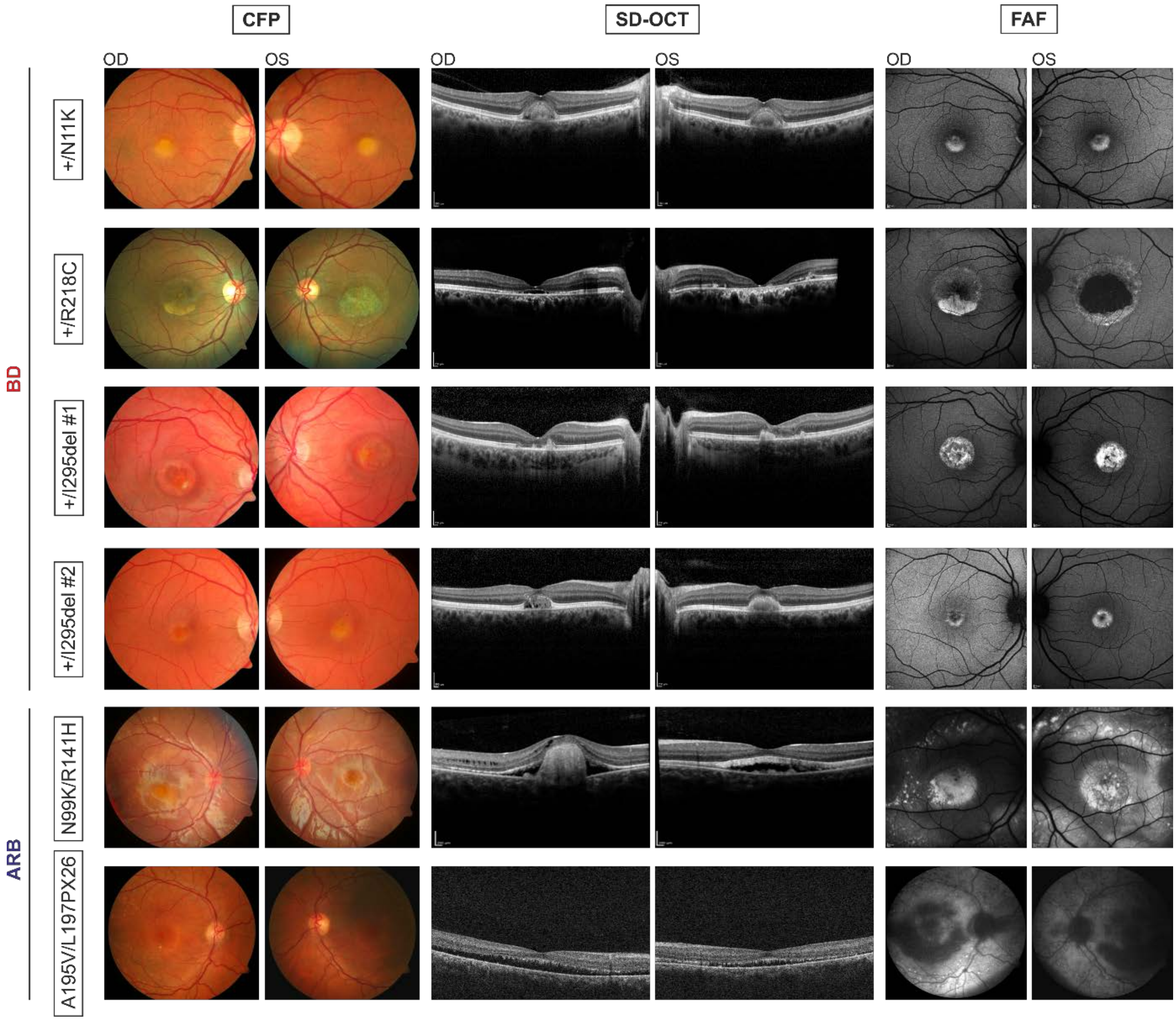

Fig. S1. Multimodal retinal imaging of patients included in the in-vitro iPSC-RPE modelling.

Color fundus images (CFP) of right (OD) and left (OS) eyes reveal the typical, bilateral, central, round, yellowish, vitelliform lesions in four patients with BD (+/N11K, +/R218C, +/I295del #1 and I295del #2). Patient with genotype +/R218C shows a pseudohypopyon stage on the right and central atrophy on the left eye. Patient with genotype +/N11K presents with a small peripheral lesion in each eye. Both ARB patients N99K/R141H and A195V/L198PX26 reveal smaller yellowish central lesions and multiple fleck-like yellowish deposits disseminated along the upper and lower vascular arcades. Foveal horizontal Spectral Domain-Optical Coherence Tomography (SD-OCT) scans of right and left eyes visualize hyperreflective subretinal material

deposits corresponding to the vitelliform material in two of the four patients with BD (+/N11K and +/I295del #1). In the right eye of +/N11K and +/I295del #1 the solid material is partially resorbed. A complete resorption of vitelliform material is seen in the right eye of +/R218C, leaving behind an optically empty subretinal cavity that is in line with the less pronounced yellowish lesions on CFP. SD-OCT further reveals central atrophy in both eyes of +/I295del #2 and in the left eye of +/R218C. Both patients with ARB genotype N99K/R141H and A195V/L197PX26 show a central likely serous detachment of the neurosensory retina as well as small subretinal deposits. Additionally, the right eye of ARB patient N99K/R141H reveals a large central accumulation of subretinal material as well as intraretinal cysts. Fundus autofluorescence (FAF) images of right and left eyes depict a distinct increase of autofluorescence corresponding to the vitelliform material in the RPE in four patients with BD (+/N11K, +/R218C right eye, +/I295del #1 and +/I295del #2). FAF of the left eye of +/R218C demonstrates hypofluorescence corresponding to the central atrophy. ARB patients N99K/R141H and A195V/L197PX26 exhibit a fleck-like increase mixed with a decreased autofluorescence along the upper and lower vascular arcades. Patient N99K/R141H reveals a pronounced central increase and A195V/L197PX26 revealed no structuralretinal changes on FAF. Please note that detailed patient information on BD patients +/Q238R and +/A243V [1] and ADVIRC patients +/V86M [2] was given previously.

Supplementary Figure S2

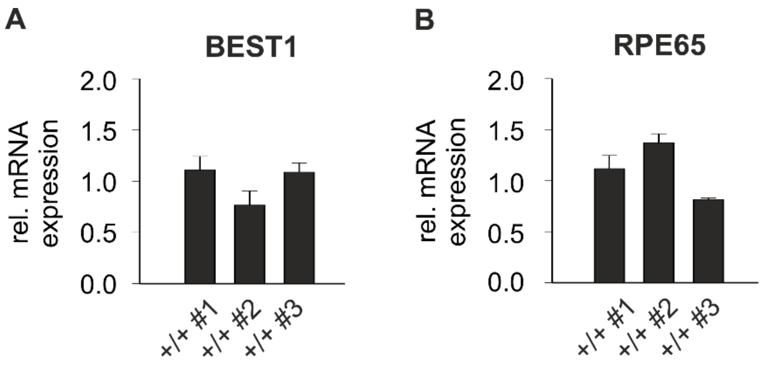

Fig. S2. mRNA expression in independent clones of control hiPSC-RPE cell lines. Quantitative real-time RT-PCR (qRT-PCR) analysis of (**A**) BEST1 and (**B**) RPE65 in independent clones of control hiPSC-RPE cell lines. RNA was extracted from hiPSC-RPE samples (n = 3) and qRT-PCR was performed in triplicates. Expression was normalized to HPRT1. Data were given as mean ± SD. A summary of hiPSC clones used in these experiments is given in **Supplementary Table S4**.

Supplementary Figure S3

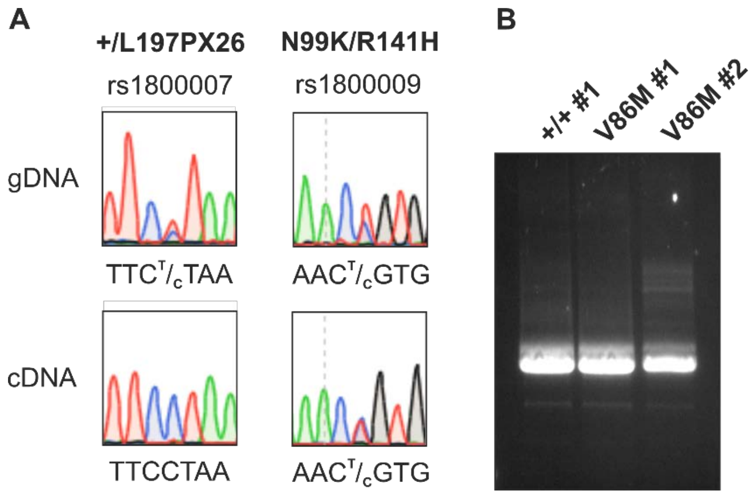

Fig. S3. Analysis of ARB-associated premature stop codon p.(L197PX26) and of splicing effect of ADVIRC-associated mutation p.(V86M).

(**A**) RNA expression of BEST1 in samples from ARB-associated hiPSC-RPE cell lines heterozygous for single nucleotide polymorphisms rs1800007 and rs1800009 located in the coding region of the *BEST1* gene. Relative expression of BEST1 was analyzed by semi-quantitative sequencing of allele-specific transcripts. Heterozygous alleles at the *BEST1* gene locus in ARB-parent +/L197PX26 and ARB-patient N99K/R141H as determined by genomic sequencing of variants rs1800007 in exon 2 (left) and rs1800009 in exon 10 (right) of the *BEST1* gene, respectively. RT-PCR analyses of the same samples reveal exclusively the presence of the normal BEST1 allele in parent +/L197PX26 whereas both alleles are present in patient N99K/R141H. (B) RT-PCR amplification of the full-length BEST1 transcript from cDNA samples obtained from hiPSC-RPEs of control (+/+ #1) and the two ADVIRC patients (V86M #1 and V86M #2) reveal the correct amplicon of 1.7 kb indicating normal splicing of exon 4. Fig. (**B**) PCR amplification of full-length BEST1 from cDNA samples produced from hiPSC-RPEs of control (+/+ #1) and the two ADVIRC patients (V86M #1 and V86M #2) revealed only the correct amplicon of 1.7 kb indicating normal splicing of exon 4.

Supplementary Figure S4

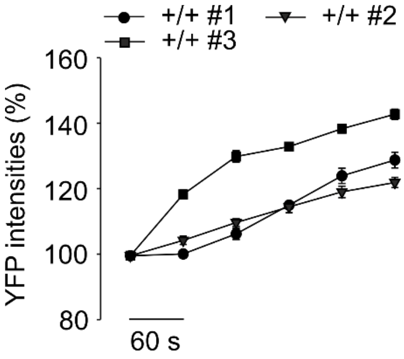

Fig. S4. BEST1-mediated anion permeability in individual control hiPSC-RPE cell lines.

Kinetic of YFP fluorescence intensities over time in hiPSC-RPEs from three independent control hiPSC-RPE cell lines (+/+ #1, +/+ #2, +/+ #3). Recordings were taken at indicated time points after initial exposure to I^- in the presence of the calcium ionophore A23187 followed by the addition of Cl^- containing solution. Mean values are given as mean ± SE from six to eighteen technical replicates per individual sample (n = 3 - 4). A summary of hiPSC clones used in experiments of Fig. S4 is shown in **Supplementary Table S4**.

Supplementary Figure S5

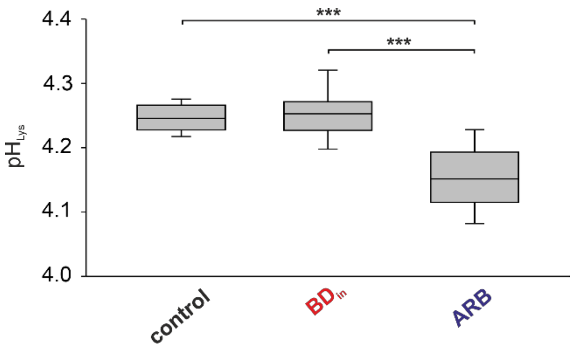

Fig. S5. Baseline lysosomal pH (pH_{lys}) in control, BD_{IN} and ARB hiPSC-RPE cell lines

Box plot showing mean values of baseline pH_{Lys} in hiPSC-RPEs from +/+ #1 and #2 (control), +/Q238R and +/I295del #2 (BD_{IN}) and the two ARB patients A195V/L197PX26 and N99K/R141H. Ratios of fluorescence excited at 385 nm and 330 nm were determined through fluorescence measurements on a plate reader using the ratiometric indicator dye LysoSensor^{TM} Yellow/Blue DND-160. Absolute values of pH_{Lys} were calculated relative to a standard curve for each cell line. Mean values are given as mean ± SD from five technical replicates per individual sample (n = 2 per phenotype). Statistical analysis was performed applying Kruskal-Wallis test for non-normal data, following post-hoc Dunn's multiple comparisons test including Benjamini-Hochberg procedure: *** = p<0.001. A summary of hiPSC clones used in experiments of Fig. S5 is shown in **Supplementary Table S4**.

References

1. Milenkovic, A.; Brandl, C.; Milenkovic, V. M.; Jendryke, T.; Sirianant, L.; Wanitchakool, P.; Zimmermann, S.; Reiff, C. M.; Horling, F.; Schrewe, H.; Schreiber, R.; Kunzelmann, K.; Wetzel, C. H.; Weber, B. H., Bestrophin 1 is indispensable for volume regulation in human retinal pigment epithelium cells. Proceedings of the National Academy of Sciences of the United States of America 2015, 112, (20), E2630-9.

2. Kellner, S.; Stohr, H.; Fiebig, B.; Weinitz, S.; Farmand, G.; Kellner, U.; Weber, B. H., Fundus Autofluorescence and SD-OCT Document Rapid Progression in Autosomal Dominant Vitreoretinochoroidopathy (ADVIRC) Associated with a c.256G > A Mutation in BEST1.Ophthalmic genetics 2016, 37, (2), 201-8.

Supplementary Table S1. Highlighted mutations in the 3D structure model of chicken Best1.

Disease	Nucleotide exchange	Amino acid exchange
BD	c.4A>G	T2A
BD	c.16A>C	T6P
BD	c.25G>A	V9M
BD	c.28G>A	A10T
BD	c.33T>G	N11K
BD	c.47C>T	S16F
BD	c.50T>G	F17C
BD	c.61C>G	L21V
BD	c.72G>T	W24C
BD	c.73C>T	R25W
BD	c.89A>G	K30R
BD	c.240C>A	F80L
BD	c.241G>A	V81M
BD	c.244C>G	L82V
BD	c.250T>G	F84V
BD	c.253T>C	Y85H
BD	c.272C>T	T91I
BD	c.274C>A	R92S
BD	c.279G>C	W93C
BD	c.286C>G	Q96E
BD	c.299T>G	L100R
BD	c.313C>G	R105G
BD	c.324C>G	S108R
BD	c.399C>G	N133K
BD	c.403G>A	G135S
BD	c.431G>A	S144N
BD	c.436_437delinsAA	A146K
BD	c.583_584insTGG	K194_A195insV
BD	c.652C>T	R218C
BD	c.662G>T	C221F
BD	c.670C>A	L224M
BD	c.679T>A	Y227N
BD	c.684C>G	D228E
BD	c.695T>A	I232N
BD	c.698C>T	P233L
BD	c.703G>A	V235M
BD	c.710C>G	T237R
BD	c.722C>A	T241N
BD	c.728C>T	A243V

Supplemmentary Table S1. cont'd

Disease	Nucleotide exchange	Amino acid exchange
BD	c.877C>A	Q293K
BD	c.880C>G	L294V
BD	c.884_886delTCA	I295del
BD	c.886A>C	N296H
BD	c.889C>G	P297A
BD	c.892T>G	F298V
BD	c.900G>C	E300D
BD	c.903T>G	D301E
BD	c.904G>A	D302N
BD	c.910_912delGAT	D304del
BD	c.914T>C	F305S
BD	c.917A>G	E306G
BD	c.920C>T	T307I
BD	c.925T>C	W309R
BD	c.929T>C	I310T
BD	c.932T>G	V311G
ARB	c.38G>A	R13H
ARB	c.122T>C	L41P
ARB	c.139C>T	R47C
ARB	c.297C>A	N99K
ARB	c.400C>G	L134V
ARB	c.422G>A	R141H
ARB	c.454C>G	P152A
ARB	c.488T>G	M163R
ARB	c.536_538delACA	N179del
ARB	c.584C>T	A195V
ARB	c.763C>T	R255W
ARB	c.821C>G	P274R
ARB	c.830C>T	T277M
ARB	c.848_850delTCT	F283del
ARB	c.949G>A	V317M
ARB	c.974T>C	M325T
ADVIRC	c.248G>C	G83D
ADVIRC	c.256G>A	V86M
ADVIRC	c.704T>C	V235A
ADVIRC	c.707A>G	Y236C
ADVIRC	c.715G>A	V239M

Supplementary Table S2. Summary of clinical information of patients with different forms of bestrophinopathies and their relatives included in the *in-vitro* iPSC-RPE modelling.

BEST1 Genotype	Age[a] [years]	Visual acutiy [logMAR]		EOG [Arden ratio]		ERG	
		OD	OS	OD	OS	OD	OS
BD							
+/N11K	59 (59)	0.2	0.2	1.1	1.3	Fullfield ERG: normal photopic amplitudes/peak times; multifocal ERG: normal	Fullfield ERG: normal photopic amplitudes/peak times; multifocal ERG: normal
+/R218C	50 (43)	1.0	1.3	1.3	1.3	Fullfield ERG: normal photopic amplitudes/peak times	Fullfield ERG: normal photopic amplitudes/peak times
+/I295del #1	50 (45)	0.3	0.1	NA	NA	Fullfield ERG: normal photopic cone response	Fullfield ERG: normal photopic cone response
+/I295del #2	17 (16)	0.7	0.4	Missing light peak	Missing light peak	NA	NA
ARB							
N99K/R141H	6 (NA)	0.1	0.1	Missing light peak	Missing light peak	Fullfield ERG: scotopic amplitudes reduced at higher flash intensities; multifocal ERG: central and paracentral amplitude reduction	Fullfield ERG: scotopic amplitudes reduced at higher flash intensities, photopic amplitudes reduced; multifocal ERG: central and paracentral amplitude reduction
+/R141H	47 (NA)	-0.1	-0.1	2.4	2.0 (normal)	Fullfield ERG: normal; multifocal ERG: normal	Fullfield ERG: normal; multifocal ERG: normal
A195V/L197PX26	26 (24)	0.3	0.3	1.3	1.3	Fullfield ERG: photopic normal latency and amplitude, scotpic normal latency, reduced amplitude; multifocal ERG: reduced central amplitudes	Fullfield ERG: photopic normal latency and amplitude, scotpic normal latency, reduced amplitude; multifocal ERG: reduced central amplitudes
+/A195V	53 (NA)	0.0	0.0	2.5	2.2	NA	NA
+/L197PX26	54 (NA)	0.0	0.0	2.7	2.2	NA	NA
ADVIRC							
+/V86M #1	50 (45)	0.2	LP	NA	NA	NA in late stage (see patient II:4 in Kellner et al., 2016)	NA in late stage (see patient II:4 in Kellner et al., 2016)
+/V86M #2	17 (16)	0.0	0.0	1.1	1.0	NA in later stage (see patient III:2 in Kellner et al., 2016)	NA in late stage (see patient II:4 in Kellner et al., 2016)

logMAR = logarithm of the Minimum Angle of Resolution; EOG = electro-oculogram; ERG = electro-retinogram; OD = right eye; OS = left eye; NA = not available; BD = Best disease; ARB = autosomal recessive bestrophinopathy; ADVIRC = autosomal dominant vitreoretinochoroidopathy; LP = light peak;
[a]) Age at harvesting of skin biopsy (age at initial diagnosis).

Supplementary Table S3. Transepithelial electrical resistance (TER) of hiPSC-RPE cell lines after five weeks growth on transwell inserts.

BEST1 Genotype	iPSC clone	TER (Ω*cm^2)
+/+ #1	MK#22	647 ± 39
	MK#27b	1194 ± 66
+/+ #2	AM#13	755 ± 83
	AM#260	884 ± 75
+/+ #3	NG	868 ± 81
+/N11K	MW#232	777 ± 73
+/R218C	MO#214	1072 ± 56
+/A243V	SK#16	855 ± 86
+/Q238R	DK#Pka	1089 ± 136
+/I295del #1	AP#187	661 ± 54
+/I295del #2	MD#18	634 ± 124
N99K/R141H	LA#29	1081 ± 79
	LA#33	1435 ± 125
+/R141H	MA#77	1160 ± 35
A195V/L197PX26	TT#178	849 ± 72
	TT#180	809 ± 60
+/A195V	PT#172	698 ± 64
	PT#176	779 ± 88
+/L197PX26	AT#166	1116 ± 34
	AT#168	960 ± 44
+/V86M #1	IS#53a	595 ± 54
	IS#53b	
+/V86M #2	JS#52a	621 ± 99
	JS#52b	

TER = transepithelial electrical resistance

Supplementary Table S4. iPSC clones generated from patient fibroblasts and allocation to tables and figures.

Figure/Table	Description	Used iPSC-clone
Fig. 2A	Confocal immunofluorescence images of monolayers from hiPSC-RPE cells	Control: MK#22; BD: MW#232, MO#214, SK#16, DK#Pka, AP#187, MD#18; ARB: LA#33, MA#77, TT#180, PT#172, AT#168; ADVIRC: IS#53a, JS#52a
Fig. 2B, C	Quantification of BEST1 and RPE65 expression by qRT-PCR from hiPSC-RPE cells	Control: MK#27b; BD: MW#232, MO#214, SK#16, DK#Pka, AP#187, MD#18; ARB: LA#33, MA#77, TT#180, PT#172, AT#168; ADVIRC: IS#53a, JS#52a
Fig. 2D	Representative Western Blot images of whole cell lysates from hiPSC-RPE cells	Control: MK#22; BD: MW#232, MO#214, SK#16, DK#Pka, AP#187, MD#18; ARB: LA#33, MA#77, TT#180, PT#172, AT#168; ADVIRC: IS#53a, JS#52a
Fig. 2E	Quantification of BEST1 protein expression.	Control: MK#22; BD: MW#232, MO#214, SK#16, DK#Pka, DK#Pkb, AP#187, MD#18; ARB: LA#29, LA#33, MA#74, MA#77, TT#178, TT#180, PT#172, PT#176, AT#166, AT#168; ADVIRC: IS#53a, IS#53b, JS#52a, JS#52b
Fig. 3C-G	Kinetic of YFP fluorescence intensities over time in hiPSC-RPE cells	Control: MK#22; BD: MW#232, MO#214, SK#16, DK#Pka, AP#187, MD#18; ARB: LA#33, MA#77, TT#178, PT#176, AT#166; ADVIRC: IS#53a, JS#52a
Fig. 3H	Averaged bar graphs showing recovery rated from initial levels of YFP quenching to maximum fluorescence signal.	Control: MK#22, AM#13, NG#1; BD: MW#232, MO#214, SK#16, DK#Pka, AP#187, MD#18; ARB: LA#33, MA#77, TT#178, PT#176, AT#166; ADVIRC: IS#53a, JS#52a
Fig. 4A	Representative Western blot images of whole cell lysates from untreated and treated control and BD_{IN} hiPSC-RPE cell lines.	Control: MK#22; BD: DK#Pka, AP#187, MD#18; ARB: LA#33, TT#180
Fig. 4B	Summary of BEST1 protein expression obtained from experiments shown in Fig4A.	BD: DK#Pka, DK#Pkb, AP#187, MD#18; ARB: LA#33, TT#180

Supplementary Table S4. cont'd

Fig. 4C	Representative Western blot images of whole cell lysates from untreated and treated control, BD_{PM} and ADVIRC hiPSC-RPE cell lines.	Control: AM#260; BD: MW#232, MO#214, SK#16; ADVIRC: IS#53b, JS#52b
Fig. 4D	Summary of BEST1 protein expression obtained from experiments shown in Fig4B.	BD: MW#232, MO#214, SK#16; ADVIRC: IS#53b, JS#52b
Fig. 5B	Box plots showing lysosomal pH in hiPSC-RPE cells.	Control: AM#13, MK#27; BD: DK#Pka, MD#18; ARB: LA#33, TT#180
Fig. 5C	Representative Western blot images of the analysis of CTSD maturation after POS feeding.	Control: MK#22; BD: DK#Pka, MD#18; ARB: LA#33, TT#180
Fig. 5D	Representative Western blot images of the analysis of CTSD maturation after treatment with CQ.	Control: MK#22; BD: DK#Pka, AP#187, MD#18; ARB: LA#29, MA#77, TT#180, AT#168, PT#172
Table S4	Transepithelial electrical resistance (TER) of hiPSC-RPE cell lines after five weeks growth on transwell inserts.	Control: MK#22, MK#27b, AM#13, AM#260, NG#1; BD: MW#232, MO#214, SK#16, DK#Pka, DK#Pkb, AP#187, MD#18; ARB: LA#29, LA#33, MA#77, TT#178, TT#180, PT#172, PT#176, AT#166, AT#168; ADVIRC: IS#53a, IS#53b, JS#52a, JS#52b
Fig. S2	Quantification of BEST1 and RPE65 expression by qRT-PCR from additional control hiPSC-RPE cells	Control: AM#13, MK#22, NG#1
Fig. S4	Kinetic of YFP fluorescence intensities over time in additional control hiPSC-RPE cells	Control: AM#13, MK#22, NG#1
Fig. S5	Box plots showing baseline lysosomal pH in hiPSC-RPE cells.	Control: AM#13, MK#22; BD: DK#Pka, MD#18; ARB: LA#33, TT#180

Supplementary Table S5. Primer pairs for BEST1 exon amplification and subsequent sequencing.

Name	Sequence (5`-3`)	Purpose
dogBest1-R	cagacctgttttccaaggcc	Amplifying of exon 10 (gDNA); sequencing of SNP rs1800009:T>C
hBest1_F_NheI	gctagcaccatgaccatcacttacacaag	Amplifying full-length BEST1 cDNA; sequencing of control and ADVIRC cDNA (+/+ #1, I295del #1 and I295del #2)
hBest1_R_MluI	acgcgtttaggaatgtgcttcatccc	Amplifying full-length BEST1 cDNA; sequencing of control and ADVIRC cDNA (+/+ #1, I295del #1 and I295del #2)
hVMD2-RT-R	gactggatcagtgtcctgctg	Amplifying of exon 10 (cDNA) Sequencing of control and ADVIRC cDNA (+/+ #1, I295del #1 and I295del #2)
mVMD2_F2	ctgtatgcctacgactggat	Sequencing of control and ADVIRC cDNA (+/+ #1, I295del #1 and I295del #2)
mVMD2_F3	agctacatccagctcatccc	Sequencing of control and ADVIRC cDNA (+/+ #1, I295del #1 and I295del #2)
N11K-BamHI-R	ggatccgtggggccagacaaagcc	Amplifying of exon 2 (gDNA), Sequencing of SNP rs1800007:T>C
N11K-EcoRI-F	gaattcatccctacaaacccccaatc	Amplifying of exon 2 (gDNA) Sequencing of control and ADVIRC cDNA (+/+ #1, I295del #1 and I295del #2)
Tu15_NEW_F3	gagaccaactggattgtcga	Sequencing of control and ADVIRC cDNA (+/+ #1, I295del #1 and I295del #2)
Tu15_NEW_F4	catgaccatcacttacacaagc	Amplifying exon 2 (cDNA), sequencing of SNP rs1800007:T>C
Tu15_Race_1H	cttgtagactgcggtgctgacg	Amplifying of exon 2 (cDNA) Sequencing of control and ADVIRC cDNA (+/+ #1, I295del #1 and I295del #2)
Tu15_Race_2	ccacggcaggttctcgtact	Sequencing of control and ADVIRC cDNA (+/+ #1, I295del #1 and I295del #2)
TU15newF6	gaagcttaaggctgtggacg	Amplifying of exon 10 (gDNA and cDNA); sequencing of SNP rs1800009:T>C

Supplementary Table S6. Primer pairs and Roche Library Probes for qRT-PCR analysis

Gene	Species	F-Primer (5`-3`)	R-Primer (5`-3`)	Roche Library Probe
BEST1	Human	agctgctatatggcgagttctt	gtgagggccagcctataaataa	4
HPRT1	Human	tgaccttgatttattttgcatacc	cgagcaagacgttcagtcct	73
RPE65	Human	gccttggaagaagatgatgg	tggcattcagaatcaggaga	68

qRT-PCR = quantitative real-time reverse transcriptase PCR
